# High-resolution 3-dimensional late gadolinium enhancement scar imaging in surgically corrected Tetralogy of Fallot: clinical feasibility of volumetric quantification and visualization

**DOI:** 10.1186/s12968-014-0076-y

**Published:** 2014-10-01

**Authors:** John Stirrat, Martin Rajchl, Lynn Bergin, David J Patton, Terry Peters, James A White

**Affiliations:** Imaging Laboratories, Robarts Research Institute, Western University, London, Ontario Canada; Division of Cardiology, Department of Medicine, Western University, Calgary, Canada; Cardiac Imaging Centre, Libin Cardiovascular Institute, University of Calgary, Calgary, Alberta Canada; Department of Pediatrics, University of Calgary, Calgary, Alberta Canada; Division of Cardiology, Department of Medicine, University of Calgary, Calgary, Alberta Canada

**Keywords:** Tetrology of Fallot, Cardiovascular MR, Scar, 3D, Late gadolinium enhancement, Congenital heart disease

## Abstract

**Background:**

The extent of surgical scarring in Tetralogy of Fallot (TOF) may be a marker of adverse outcomes and provide substrate for ventricular arrhythmia. In this study we evaluate the feasibility of high resolution three dimensional (3D) late gadolinium enhancement (LGE) cardiovascular magnetic resonance (CMR) for volumetric scar quantification in patients with surgically corrected TOF.

**Methods:**

Fifteen consecutive patients underwent 3D LGE imaging with 3 Tesla CMR using a whole-heart, respiratory-navigated technique. A novel, signal-histogram based segmentation technique was tested for the quantification and modeling of surgical scar. Total scar volume was compared to the gold standard manual expert segmentation. The feasibility of segmented scar fusion to matched coronary CMR data for volumetric display was explored.

**Results:**

Image quality sufficient for 3D scar segmentation was acquired in fourteen patients. Mean patient age was 32.2 ± 11.9 years (range 21 to 57 years) with mean right ventricle (RV) ejection fraction (EF) of 53.9 ± 9.2% and mean RV end diastolic volume of 117.0 ± 41.5 mL/m^2^. The mean total scar volume was 11.1 ± 8.2 mL using semi-automated 3D segmentation with excellent correlation to manual expert segmentation (r = 0.99, bias = 0.89 mL, 95% CI -1.66 to 3.44). The mean segmentation time was significantly reduced using the novel semi-automated segmentation technique (10.1 ± 2.6 versus 45.8 ± 12.6 minutes). Excellent intra-observer and good inter-observer reproducibility was observed.

**Conclusion:**

3D high resolution LGE imaging with semi-automated scar segmentation is clinically feasible among patients with surgically corrected TOF and shows excellent accuracy and reproducibility. This approach may offer a valuable clinical tool for risk prediction and procedural planning among this growing population.

## Background

Tetralogy of Fallot (TOF) is a cyanotic congenital heart defect affecting approximately 1 in 3,600 live born infants [1]. Characteristic features include a perimembranous ventricular septal defect (VSD) and a varying degree of infundibular stenosis that require surgical correction early in life. However, such interventions are inherently associated with some degree of iatrogenic scar that contribute to regional aneurysmal dilation, systolic dysfunction and offer substrate for the development of ventricular arrhythmia [2,3]. While markers of future cardiovascular events such as the severity of pulmonic insufficiency [4] have been explored, the extent of initial surgical disruption and scar burden may be a valuable prognosticator [5]. Given the increasing incidence of patients with surgically corrected congenital heart disease [6], the development and validation of tools aimed at spatially characterizing and quantifying myocardial scar in this population are of clinical importance.

Cardiovascular magnetic resonance (CMR) is an established and preferred imaging modality for the evaluation of patients with congenital heart disease [7]. However, the potential value of late gadolinium enhancement (LGE) imaging to characterize the effect of surgical disruption has only recently been explored among patients with TOF [5,8-10]. The literature demonstrates the capacity of this technique to identify surgical scarring of the right ventricle (RV) and interventricular septum using conventional, two dimensional (2D) LGE techniques. Volumetric quantification of this scar has proved particularly challenging however, due to its thin and complex architecture. As potential exists for scar segmentation to function as both a prognostic tool [11-13] and therapeutic roadmap [14] for arrhythmia ablation, its translation to this patient population is of clinical interest. To date, such efforts have been limited to subjective visual scoring of scar distribution.

Here we test the feasibility of high resolution, isotropic 3D LGE CMR imaging for the characterization of surgical scar in patients with surgically corrected TOF. In addition, we describe a novel segmentation technique for rapid image processing and volumetric modeling.

## Methods

### Subjects and CMR acquisition

Fifteen consecutively identified patients with surgically corrected TOF were recruited from an outpatient adult congenital heart disease clinic. Patients were required to be ≥18 years of age and have no contraindications to contrast-enhanced CMR, inclusive of a glomerular filtration rate ≤45 ml/min/1.73 m^2^. All patients provided written informed consent and the study was approved by the Research Ethics Board of Western University.

Imaging was performed using a 3-Tesla CMR scanner (TRIO, Siemens Medical Systems, Erlangen, Germany) equipped with a 32-element body surface receiver coil. The imaging protocol was composed of four sequential procedures; Cine imaging, 3D contrast enhanced magnetic resonance angiography (MRA), standard 2D LGE imaging (10 minutes post contrast administration), followed by 3D LGE imaging (20-25 minutes post contrast administration). Cine images were acquired using a steady-state free precession based pulse sequence in serial short-axis slices from the atrioventricular annulus to the apex at 10-mm intervals, as well as in long-axis orientations (slice thickness 6 mm, gap 4 mm, echo time 1.5 ms, repetition time 3.0 ms, flip angle 50°). A 3D whole heart, inversion recovery gradient echo pulse sequence with a respiratory navigator pulse placed over the right hemidiaphragm was used to obtain both an early (coronary enhanced) and late (scar enhanced) dataset, as previously described by our group [15]. Typical imaging parameters were: echo time 1.3 ms; flip angle 20°; integrated parallel acquisition technique 2, end-expiratory respiratory navigator acceptance window 3.5 mm, phase encoding direction anterior posterior. The voxel size was maintained at 1.3 × 1.3 × 1.3 mm (interpolation to 0.65 mm for reconstruction) in all subjects fat saturation was employed to suppress pericardial fat signal. Imaging volumes were prescribed in the transverse plane (anterior-posterior phase encoding direction) from above the aortic arch to below the most inferior aspect of the heart (slab thickness 140-160 slices) based on multiplanar scout images. Adjustment of trigger delay and segment number was performed to maintain image acquisition between the onset and termination of cardiac standstill, as determined from the 4-chamber cine. The prescribed segment number ranged from 35 to 45 per cycle, corresponding to acquisition windows of 110 to 150 msec.

For the contrast-enhanced MRA, an intravenous infusion of 0.2 mmol/kg gadolinium (Gadovist®, Bayer Inc., Toronto, Ontario, Canada) was administered at 0.3 ml/s, followed by 40 ml of saline at the same rate. Imaging was initiated 20 seconds following infusion onset, as previously described [16].

Standard 2D LGE was performed 10 minutes following infusion completion using an inversion-recovery gradient echo pulse sequence in sequential short axis imaging from a slice location cranial to the pulmonary valve through to the apex, in standard LV long axis views, and using an RV inflow-outflow view. Typical imaging parameters; 8 mm slice thickness, 2 mm gap, 1.6 × 1.3 mm in-plane resolution.

Finally, a delayed (scar enhanced) 3D LGE dataset was acquired 20 to 25 min following infusion completion using adjustment of the inversion time (TI) to provide optimal myocardial signal suppression (typical range 240 to 270 ms), as previously described [17].

### CMR image interpretation

Serial short-axis cine images were evaluated using semi-automated commercially available software (CMR^42^, Circle International, Calgary, Alberta, Canada) to obtain end-diastolic and end-systolic volumes of the left and right ventricles. For both ventricles, a long-axis view was cross-referenced to determine the basal inclusion of volumes throughout the cardiac cycle, inclusive of the outflow tracts. Papillary and trabecular muscle architecture were excluded from the blood pool for volumetric analysis. Contrast-enhanced MRA and matched scar-enhanced datasets were visually scored for image quality and interpretability using a 5-point scale, as follows; 0 = uninterpretable, 1 = unacceptable due to artifact, 2 = acceptable with artifact, 3 = good, 4 = excellent.

**Figure 1:**
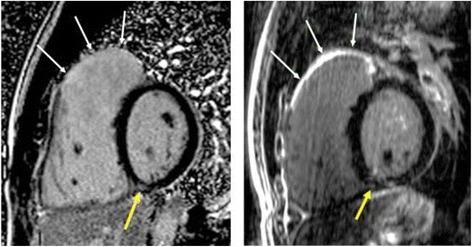
**Comparison between (left) 2-dimensional late gadolinium enhancement (LGE) imaging (typical voxel size 2.9 × 1.9 × 6 mm) and (right) corresponding high-resolution 3D LGE imaging (voxel size 1.3 × 1.3 × 1.3 mm) in a patient with surgically corrected Tetralogy of Fallot.** White arrows indicate region of surgical infundibulotomy scar with improved characterization of the thin surgical scar being apparent using 3D LGE imaging. Yellow arrows indicate inferior RV insertion site fibrosis.

**Figure 2:**
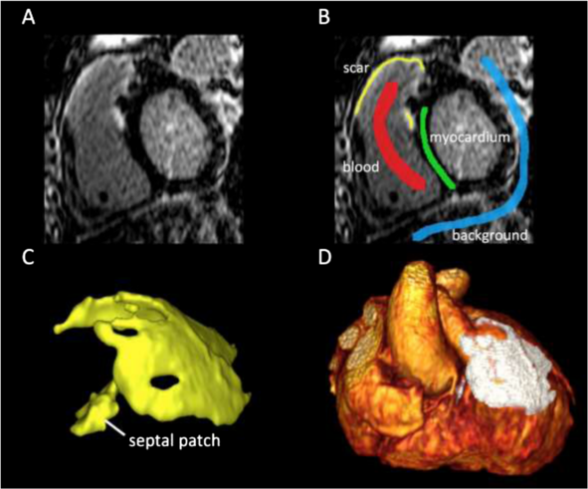
**Semi-automated scar segmentation algorithm using custom software. (A)** Raw image dataset of 3D-LGE shown in the axial plane at the level of the right ventricular outflow tract. **(B)** A brush stroke label is applied over a region of scar (yellow), blood pool (red), myocardium (blue), and the background tissue (gray). **(C)** 3D scar segmentation results shown in a volume-rendered format. Infundibulotomy and septal patch scar are both clearly visible. **(D)** Final fused 3D-Cardiac MRA/Scar image shown in volume-rendered format.

All 2D and 3D LGE datasets were visually scored for the presence or absence of RV outflow tract and septal patch related scar (Figure 1). Volumetric scar segmentation of conventional 2D scar-enhanced datasets was performed using manual planimetry. This approach was required as manual border tracing (required for threshold-based analysis) was found to be inaccurate and frequently contaminated by inclusion of epicardial fat and/or blood pool, due to the thin and complex RV architecture. Importantly, phase reconstructed 2D LGE images were used for this analysis as (in our clinical experience) this provides improved visualization of scar within the RV wall. For 3D LGE scar segmentation, a novel, semi-automated software that employs a Hierarchical Max Flow algorithm was employed, as previously described [18]. This method uses a brush tool to obtain a single sampling of four distinct regions (Figure 2); normal myocardium, blood pool, scar and background. Signal-intensity histograms from these respective regions are used to classify all remaining voxels into one of these categories using a probabilistic approach, as described by Rajchl *et al.* [19,20]. This algorithm employs a region-ordering constraint that exploits prior knowledge of rudimentary cardiovascular anatomy. This avoids the need for manual boundary identification, and largely eliminates the need for manual correction of segmented data. The time duration for computer-assisted segmentations (CAS) was recorded for all 3D image datasets.

To facilitate accuracy in the assessment of the semi-automated scar segmentation technique, as well as to provide a benchmark for segmentation efficiency, all datasets were manually segmented by a CMR expert blinded to patient information (JAW). This was performed by manual brush tool labeling of enhanced voxels using an identical 3D multiplanar reconstruction (MPR) interface, with time required for segmentation noted again. This was chosen as the best available gold standard. CAS-based and gold standard manual segmentation total scar volumes were compared for both accuracy and image-processing time (efficiency). In addition, an agreement evaluation for the spatial accuracy of voxel labeling (scar versus not scar) was performed using standardized accuracy metrics commonly reported for segmentation validation. The Dice Similarity Coefficient was used to express the percentage of all voxels assigned the same binary value, and the average Root-Mean squared Surface Error (RMSE) was used to express the average Euclidean distance between the two generated surfaces.

Finally, CAS-based scar segmentation results were fused to the spatially matched 3D contrast-enhanced MRA datasets (inherently spatially co-registered) for volumetric presentation using an interactive 3D volume rendered display (OsiriX, Version 4.1.2), as shown in Figure 2D.

### Intra- and inter-observer reproducibility

Two blinded interpreters (J.S. and M.R.) performed CAS-based scar segmentation on all 3D scar-enhanced datasets to determine both intra- and inter-observer reproducibility of this technique for the measurement of total scar volume. Both users also performed manual planimetry on all 2D LGE datasets to calculate the intra- and inter-observer variability of this conventional technique.

### Statistical analysis

Data are expressed as mean values ± standard deviation. Comparisons of continuous variables (segmentation time) were performed by means of a 2-tailed *t*-test, as indicated in the Results section. A p-value ≤0.05 was considered significant. Linear regression and Bland-Altman analyses were performed to determine the correlation and difference in measured scar volume between CAS and manual segmentation, as well as intra- and inter-observer variability. Statistical analyses were performed using a commercially available software program (GraphPad Prism, Version 6.0, GraphPad Software, San Diego, California).

## Results

### Baseline characteristics

Baseline clinical characteristics are shown in Table 1. The mean age was 32.2 ± 11.9 years (range 21 to 57 years), and eight patients (57%) were female. The average time from TOF surgical repair was 30.3 ± 11.9 years (range 17 to 49 years).

**Table 1:** **Non-CMR baseline patient characteristics, presented for the total population**

**Variable**	**Total cohort**
Age (years)	32.2 ± 11.9
Female sex	8 (57%)
Caucasian	14 (100%)
Systolic BP (mmHg)	120.1 ± 13.9
Diastolic BP (mmHg)	76.3 ± 8.1
Heart rate (beats/min)	68.7 ± 9.6
GFR (ml/min/1.73 m^2^)	103.9 ± 23.9
ECG	
LBBB	0 (0%)
RBBB	8 (57%)
QRS duration (msec)	138.3 ± 28.2
Years since surgery	30.3 ± 11.9
NYHA class	
Class I	12 (86%)
Class II	2 (14%)
Medications	
ACE inhibitor/ARB	2 (14%)
ASA	1 (7%)
Beta-blocker	2 (14%)
Digoxin	1 (7%)
Diuretic	1 (7%)

Continuous data are expressed as mean ± SD, categorical data as n MI = Myocardial infarction, GFR = Glomerular Filtration Rate; LBBB, left bundle-branch block; NYHA = New York Heart Association; ICD = Implantable cardiac defibrillator, CRT = Cardiac resynchronization therapy; ACE = angiotensin-converting enzyme; ATII = angiotensin II.

Baseline CMR characteristics are listed in Table 2. The mean left ventricular ejection fraction (LVEF) was 63.7 ± 8.0%, and the mean right ventricular ejection fraction (RVEF) was 53.9 ± 9.2%. The mean RV end diastolic volume (EDV), indexed to body surface area, was 117.2 ± 40.0 mL/m^2^.

**Table 2:** **CMR parameters presented for total population**

**Variable**	**Total cohort**
**Chamber volumes**	
RV EF (%)	53.9 ± 9.2
RV ESVi (mL/m^2^)	55.2 ± 26.3
RV EDVi (mL/m^2^)	117.0 ± 41.5
LV EF (%)	63.7 ± 8.0
LV ESVi (mL/m^2^)	25.1 ± 10.5
LV EDVi (mL/m^2^)	68.6 ± 21.9
**Scar quantification (3D CAS)**	
Total scar volume (ml)	11.1 ± 8.2

Continuous data are expressed as mean ± SD. RV = Right ventricle; EF = Ejection fraction, ESVi = End-diastolic volume index, EDVi = End-systolic volume index, LV = Left ventricle, CAS = Computer Assisted Segmentation.

Three dimensional contrast-enhanced and scar-enhanced image datasets were obtained in all 15 patients with mean heart rate during image acquisition determined to be 68 ± 8 (range 56 to 85) beats/min. All patients were in sinus rhythm at the time of image acquisition. Acquisitions ranged from 649 ± 72 cardiac cycles with corresponding mean image acquisition times of 7.1 ± 1.4 mins for coronary MRA and 7.2 ± 1.6 mins for LGE. Combined image quality was scored as acceptable (quality score ≥ 2 out of 4) in 14 of 15 patients (93%). The number of patients with quality scores of 1, 2, 3, and 4 were N = 0, 1, 4, and 10 respectively for the 3D MRA, and N = 1, 2, 6, and 5 for the 3D LGE datasets, respectively. One patient exhibited severe motion-related artifacts on the scar-enhanced data acquisition due to coughing and this data was therefore excluded due to an inability to perform analysis.

### 3D contrast-enhanced and scar-enhanced imaging

The mean image acquisition times for contrast-enhanced and scar-enhanced datasets were 7.9 ± 2.6 and 7.1 ± 2.5 minutes, respectively. Mean quality scores were 3.6 ± 0.6, and 3.2 ± 0.7 out of 4, respectively. The quality scores of the excluded patient were 4 and 1 out of 4, respectively, as the patient coughed throughout scar-enhanced acquisition.

**Figure 3:**
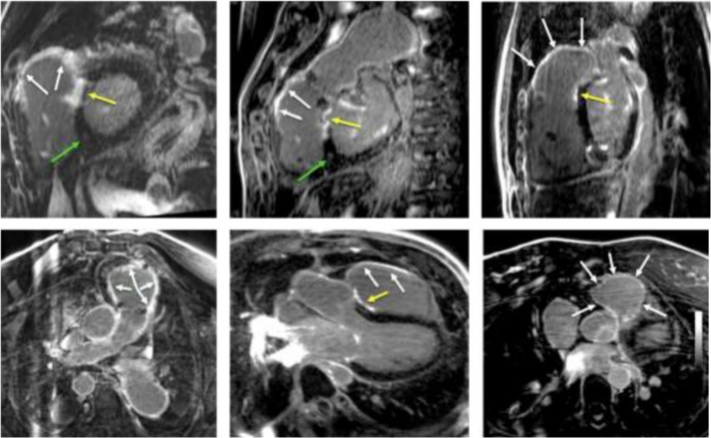
**Case examples of high-resolution, isotropic 3D late gadolinium enhancement (LGE) imaging in patients with surgically corrected Tetralogy of Fallot, presented in representative views using multi-planar reformatting.** White arrows: Surgical infundibulotomy scar. Yellow arrows: Surgical ventricular septal repair scar. Green arrows: Acquired right ventricular insertion site fibrosis.

Visual analysis of 3D scar-enhanced datasets showed clear evidence of myocardial scar in the right ventricular outflow tract (RVOT) (infundibulotomy site) of all 14 patients (100%), and of the basal interventricular septum (VSD repair site) in 12 of 14 patients (86%). Typical case examples are provided in Figure 3. Corresponding 2D LGE image scoring showed clear evidence of RVOT scar in 12/14 patients (86%) and basal septal scar in 3/14 patients (21%). In contrast, non-ischemic fibrosis of the RV insertion site was scored as present in 6/14 patients (43%) on 3D LGE, and 11/14 patients (79%) on 2D LGE imaging. This latter pattern of LGE, when visualized, was excluded from 3D segmentation for two reasons; i) signal histograms of surgical (dense) scar were found to be unique to non-surgical (low signal/patchy) scar and required separate initialization for signal profile based segmentation, and ii) the primary aim of the study was to evaluate methodology for surgical scar quantification and modeling, in particular towards its translation for image-guided therapies, such as the ablation of scar re-entry tachyarrhythmia.

### 3D scar segmentation and image fusion

3D scar segmentation was successfully performed in all 14 patients using both CAS and manual expert segmentation. Total segmentation time (from initiation of image file upload to satisfactory generation of a final 3D segmented scar model) using each respective technique was 10.1 ± 2.6 min (range 5 to 14 minutes) versus 45.8 ± 12.6 min (range 35 to 70 minutes) (p < 0.0001), representing a 4-fold reduction in image processing time using the CAS approach.

**Figure 4:**
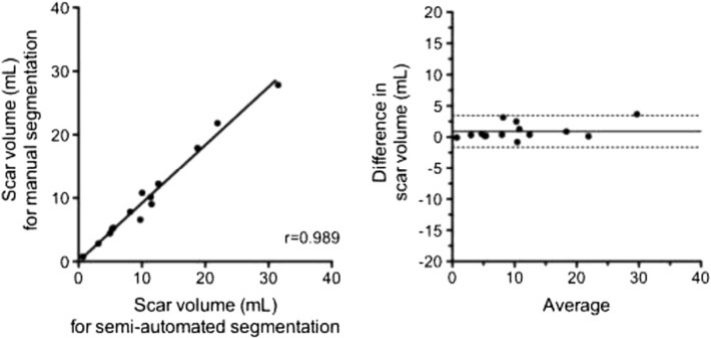
**Linear regression (left) and Bland-Altman (right) analyses for accuracy of semi-automated scar segmentation algorithm versus the gold standard of manual segmentation (N = 14).** For linear regression analysis, the Pearson correlation coefficient (r) is shown, and for Bland-Altman analysis the bias is shown with a solid line (95% confidence interval shown by dashed line).

**Figure 5:**
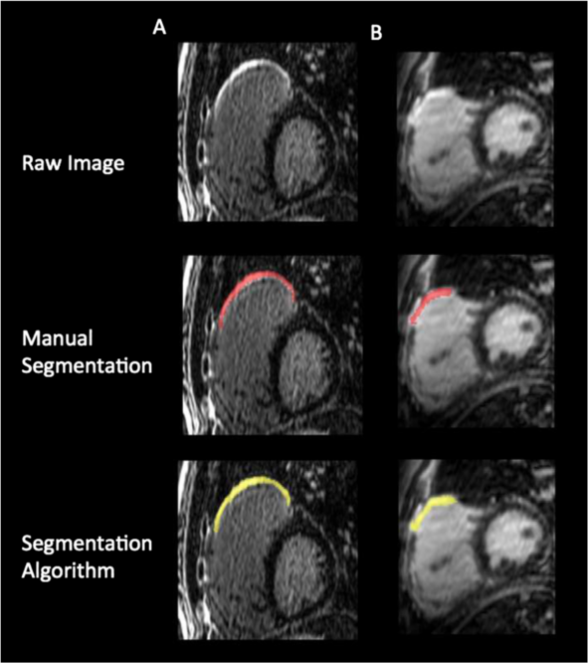
**Representative sagittal slices obtained by 3D LGE imaging in two patients (A and B) with Tetralogy of Fallot.** Thin-slab, multiplanar reformatting is shown at the level of the surgical infundibulotomy. Results of manual expert segmentation (red) and semi-automated segmentation (yellow) are shown below.

The mean total scar volume obtained using the CAS-based technique was 11.1 ± 8.2 mL. This showed excellent correlation to total scar volumes generated by manual expert segmentation with a correlation coefficient of 0.99 (Figure 4), representative patient examples being shown in Figure 5. Bland-Altman analysis showed excellent agreement with a non-significant bias of +0.89 mL (95% CI -1.7 to 3.4) (Figure 4). Voxel-based spatial accuracy metrics for CAS-based scar segmentation were excellent with a Dice similarly coefficient of 0.71 ± 0.04 and RMSE of 0.72 ± 0.15 mm.

**Figure 6:**
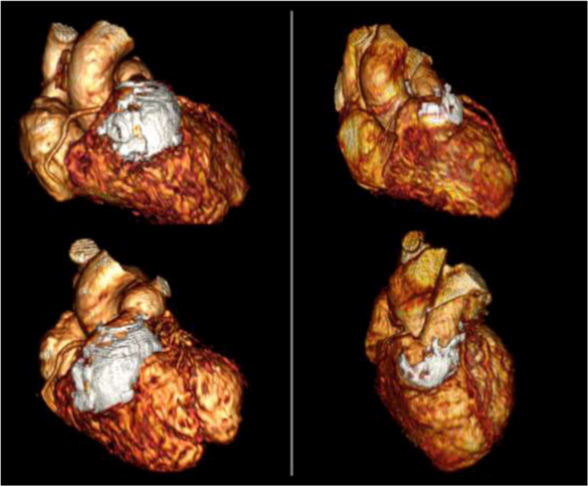
**Examples of fused 3D MRA/3D scar imaging in patients with surgically corrected Tetralogy of Fallot.** Segmented surgical scar tissue is shown in white.

All 14 cases underwent successful fusion of segmented 3D scar to spatially matched 3D MRA datasets. Case examples of volumetric display are provided in Figure 6.

### Intra- and inter-observer reproducibility

**Figure 7:**
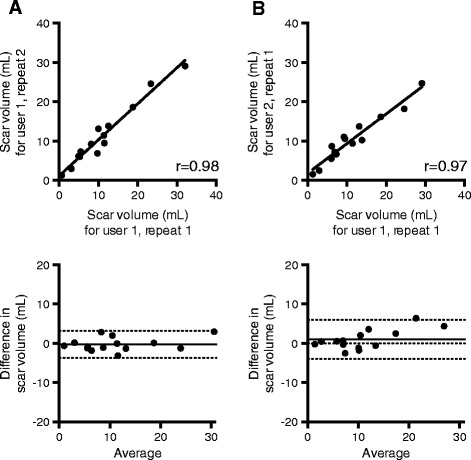
**Intra-observer (A) and inter-observer (B) variability testing for semi-automated scar quantification.** For linear regression analysis (top), the Pearson correlation coefficient (r) is shown, and for Bland-Altman analysis (bottom) the bias is shown with a solid line (95% confidence interval shown by dashed line).

Intra-observer reproducibility was excellent [r = 0.98, bias = -0.2, 95% CI -3.677 to 3.235] with similar inter-observer reproducibility [r = 0.97, bias = 1.008, 95% CI -3.914 to 5.929] (Figure 7). No significant difference in segmentation time was appreciated between the two users (10.1 ± 2.7 vs. 9.6 ± 2.1, p = 0.76).

### Comparison to 2D LGE imaging

**Figure 8:**
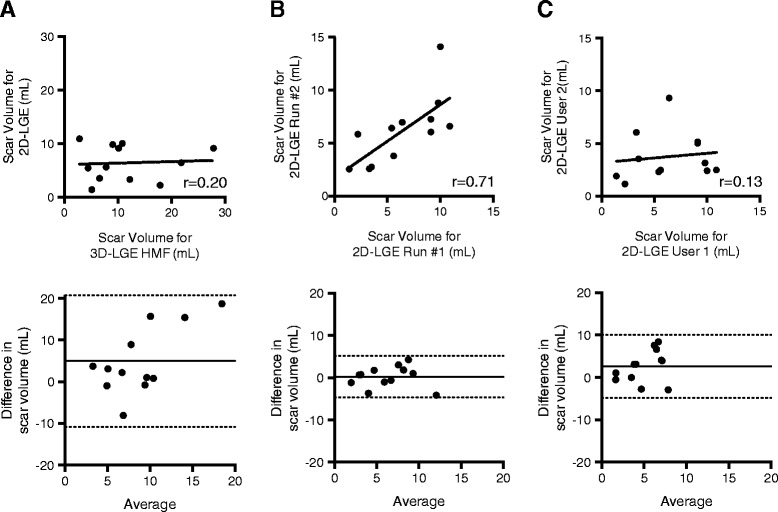
**Comparison of 2D manual segmentation, and 3D semi-automated segmentation results (left panel, A), and reproducibility of 2D manual segmentation (right).** Intra-observer **(B)** and inter-observer **(C)** variability testing results for 2D semi-automated scar quantification are shown. For linear regression analysis (top), the Pearson correlation coefficient (r) is shown, and for Bland-Altman analysis (bottom) the bias is shown with a solid line (95% confidence interval shown by dashed line).

To compare 3D scar quantification to conventional (lower resolution, anisotropic) 2D scar imaging, manual planimetry of surgical scar on the latter (seen in 12 of 14 datasets, 86%) was performed. Mean segmentation volumes were then compared to corresponding 3D LGE scar analysis. As shown in Figure 8A, 2D scar segmentation values poorly correlated with 3D scar volumes, producing a low correlation coefficient (r) of 0.2. Intra- and inter-observer variability of 2D scar quantification was substantially poorer than for 3D scar quantification (Figure 8B and C).

## Discussion

This study demonstrates the clinical feasibility of high resolution volumetric scar imaging in patients with surgically corrected TOF. The quality of this imaging technique supports the use of rapid segmentation tools to provide accurate and reproducible estimates of total scar burden and to generate volumetric models of scar distribution. As the former is of current interest in the investigation of risk prediction, and the latter affords spatial registration of scar within complex anatomic geometry, this novel technique may be of clinical value among this population.

CMR is an established and preferred imaging modality for the assessment of patients with TOF and other congenital heart diseases. Its capacity to simultaneously evaluate cardiac function, 2D and 3D morphology, and non-invasively quantify hemodynamics without exposure to ionizing radiation, has made this a commonly performed diagnostic test within this population [7]. While the use of LGE imaging for evaluations of myocardial scar may offer incremental value in these patients, several challenges exist for its clinical adoption using conventional techniques [5,8-10]. The native RV tissue is characteristically thin, only a few millimeters in thickness, and remains so among many patients with early surgical correction of TOF. Surgical intervention leads to marked thinning of the free wall as it is replaced by patch material and surrounding scar. Both native RV tissue and iatrogenic scar are juxtaposed to pericardial adipose stores and the posterior sternum. Within the context of larger and anisotropic voxels, ample opportunity is provided for partial volume effects to limit the discrimination of scar signal from adjacent tissue signal on 2D LGE images. Indeed, no study to date has attempted signal-threshold based quantification of RV scar among this patient population. An incremental contributor to this challenge is that the architecture of the RVOT is complex and does not maintain co-axial position to any single imaging plane, exaggerating partial volume influences for anisotropic 2D techniques. In contrast, the isotropic 3D imaging technique described provides a 10-fold reduction in voxel volume and effectively eliminates adjacent fat signal through application of a fat-saturation pulse. In combination, this significantly improves visualization of surgical scar (Figure 8) and affords the rapid and reproducible semi-automated segmentation of myocardial scar in this challenging patient population.

A prognostic role for myocardial scar quantification is emerging among patients with a variety of cardiomyopathy states. Numerous studies have recognized that the presence and volume of myocardial scar predicts the occurrence of ventricular arrhythmias among patients with ischemic cardiomyopathy [21-23], dilated cardiomyopathy [22], hypertrophic cardiomyopathy [24], and sarcoidosis [25,26]. Two studies to date have described the use of LGE imaging to examine the extent of surgical injury in TOF, both being limited to a visual-based scoring [5,10]. In a study by Babu-Naryan *et al*., adult patients with surgically corrected TOF received conventional 2D LGE imaging at 1.5 Tesla with the presence and distribution of surgical scar visually scored using a standardized RV segmental model. In that study, the burden of RV scar was associated with greater New York Heart Association (NYHA)-based symptom burden, reduced maximal exercise capacity (VO_2_), RV systolic dysfunction, and elevated atrial natriuretic peptide levels [5]. Most importantly, RV scar scores were independently associated with the occurrence of future arrhythmias. In a subsequent study by Wald *et al.* the segmental presence of surgical scar was associated with a reduction in systolic function of both the RVOT and of the RV globally. These were in turn associated with a reduction in exercise capacity and an increased prevalence of sustained ventricular tachycardia [10].

The potential of volumetric scar segmentation models to be used to guide catheter-based therapies in patients with adult congenital heart disease is of particular interest. Using the currently described techniques, spatially accurate 3D myocardial scar models can be generated that are inherently co-registered to the volumetric imaging datasets currently accepted by clinically available navigation tools. This provides a unique opportunity to guide catheter-based procedures, such as ventricular arrhythmia ablation, using substrate-based models – a new paradigm for invasive arrhythmia therapeutics. Such methodology is a priority for future work.

### Limitations

The study was not designed to evaluate the impact of RV scar extent on patient symptoms, exercise capacity or clinical events; endpoints that have been evaluated within larger studies [5,10]. As a small, single center feasibility study, we recommend that this technique be tested by other referral centers, and be evaluated for its capacity to predict important clinical outcomes. We also recognize that, due to labor intensity of manual segmentation, only one expert interpreter performed this task. Accordingly, reproducibility of the gold standard was not established.

Comparison of 3D scar quantification with 2D scar quantification herein was primarily undertaken to demonstrate the challenges of RV scar segmentation using conventional 2D LGE images. As optimization of a 2D imaging approach was not the study’s focus, it is possible that other pathways towards the improvement of 2D acquisitions could be explored, such as; reduction in slice thickness, inter-slice gap, and the application of fat-suppression techniques. However, all such adaptations may not adequately address a need to provide imaging data sufficiently robust to allow for semi-automated scar segmentation, and do not provide matched MRA data that facilitates rapid image fusion for anatomic spatial registration.

In this sentinel cohort study we did not identify focal scar (i.e.: amenable to segmentation) in regions remote to the primary surgical sites. However, such findings can be observed in this population and are therefore not sufficiently studied by this pilot study. Segmentation times might also be affected by the need to capture such regions. Patchy mid-wall fibrosis of the RV insertion site was not segmented for 2 reasons; i) the clinical relevance of this finding for the navigation of invasive procedures is uncertain, and ii) its signal characteristics are unique to that of surgical scar. The latter highlights a limitation of all current segmentation algorithms for their application in this population, in that unique thresholds must be employed for the capture of disparate fibrosis phenotypes.

## Conclusions

High resolution, 3D LGE imaging of myocardial scar in surgically repaired TOF is clinically feasible and affords accurate and reproducible estimates of total scar volume as well as volumetric modeling of scar distribution. Given growing clinical interest in scar metrics for the prediction of arrhythmia risk, and minimally invasive therapies aimed at their treatment, this novel imaging technique may be well suited as a clinical tool to assist in the care of this expanding population.

## Abbreviations

2D: Two dimensional
3D: Three dimensional
CAS: Computer assisted segmentation
CI: Confidence interval
CMR: Cardiovascular magnetic resonance
EDV: End diastolic volume
EF: Ejection fraction
LGE: Late gadolinium enhancement
LV: Left ventricle
MPR: Multiplanar reconstruction
MRI: Magnetic resonance imaging
MRA: Magnetic resonance angiography
NYHA: New York Heart Association
RMSE: Root mean squared error
RV: Right ventricle
RVOT: Right ventricular outflow tract
TI: Inversion time
TOF: Tetralogy of Fallot
VSD: Ventricular septal defect
